# Draft of Zucchini (*Cucurbita pepo* L.) Proteome: A Resource for Genetic and Genomic Studies

**DOI:** 10.3389/fgene.2017.00181

**Published:** 2017-11-21

**Authors:** Giuseppe Andolfo, Antimo Di Donato, Reza Darrudi, Angela Errico, Riccardo Aiese Cigliano, Maria R. Ercolano

**Affiliations:** ^1^Department of Agriculture Sciences, University of Naples ‘Federico II’, Naples, Italy; ^2^Department of Horticulture, College of Agriculture and Natural Resources, University of Tehran, Karaj, Iran; ^3^Sequentia Biotech Eureka, Barcelona, Spain

**Keywords:** RNA-seq, proteome, *Cucurbita pepo*, *R-*genes, orthology

## Introduction

The Cucurbitaceae family is the second most large horticultural family in terms of economic importance after Solanaceae. It includes several important crops, such as melon (*Cucumis melo*), watermelon (*Citrullus lanatus*), cucumber (*Cucumis sativus*) and many *Cucurbita* species with edible fruits (Jeffrey, 1980). The genus *Cucurbita* (2x = 2n = 40), originated in the Americas, encompasses three economically important crop species such as *Cucurbita pepo, Cucurbita moschata*, and *Cucurbita maxima*, cultivated throughout temperate, sub-tropical, and tropical regions (Wang et al., 2011). *Cucurbita pepo* includes a wide assortment of varieties and cultivars, known for their unique fruit shape and color and appreciated for their culinary properties. Among different species of this genus, *Cucurbita pepo* have the greatest monetary value (Paris, 2008). Botanical classification based on allozyme variation recognized three subspecies in this species including: *pepo, ovifera* (syn. *texana*), and *fraterna*. Paris (1986) classified edible-fruited *C. pepo* into eight cultivar-groups: Acorn, Crookneck, Scallop, and Straightneck that belong to subsp. *ovifera* and Pumpkin, Zucchini, Cocozelle, and Vegetable Marrow that belong to subsp. *pepo* (Paris, 2010). The genome size of *Cucurbita* spp. is approximately 500 Mb (Arumuganathan and Earle, 1991). Recently, a high-quality draft of *C. pepo* (subsp. *pepo* cultivar-group Zucchini) genome with a sequences length of about 265 million base pairs (Mbp) was made available on CucurbiGene database as well as several *C. pepo* transcriptomes have been explored (Blanca et al., 2011; Wyatt et al., 2015; Vitiello et al., 2016; Xanthopoulou et al., 2016, 2017; Montero-Pau et al., 2017). However, still little is known about the genetic diversity of this noteworthy crop and even less has been done to explore its proteome. High-throughput sequencing of transcriptomes has opened the way to study the genetic and functional information stored within any organism at an unprecedented scale and speed.

Transcriptome generation through RNA sequencing (RNA-seq) is a technology that can be used in the high resolution and broad dynamic range gene expression studies and in the simultaneous understanding of the genes function (Wang et al., 2009). Basically, the protein-coding genes function is inferred by the analysis of structure, function and evolution of the proteins they encode (Guo, 2013). For the characterization of unannotated proteins, can result particularly useful to undertake orthology analysis. Proteome data are important resources for having an overall genome vision but at the same time achieving a high level of accuracy in comparative studies (Andolfo et al., 2014a). To this end, we sequenced and assembled the first transcriptome of zucchini cultivar “True French,” founder of important pathogen resistant commercial varieties and to harness the full potential of such data we performed also an high-quality proteome annotation. A total of 33,966 protein sequences were predicted, functionally annotated and compared to cucumber, melon, watermelon and Arabidopsis proteomes. In addition, disease resistance (*R*) gene family was finely characterized and several specie-specific *R*-genes expansion was detected in *C. pepo*.

## Value of the data

- The transcriptome obtained can be used as reference for gene expression analysis. Genetic and breeding studies will be enhanced by tools and insights developed from this resource.
- The transcriptome sequence data were assembled and annotated to create a *C. pepo* reference proteome for future genomic works in this species.
- Zucchini is an important crop that lack of molecular genetics information. The transcriptome and proteome released will drive new discovery to understand complex agronomic traits and to identify novel resistance gene loci.
- The predicted proteome and comparative dataset provided will facilitate the understanding of evolutionary mechanisms of expansion/contraction of important gene families, such as resistance genes, in *Cucurbita* spp.

## Experimental design, materials and methods

### Plant material, total RNA extraction and quality control, library preparation and RNA-seq

Plants of *Cucurbita pepo* subsp. *pepo* cultivar-group Zucchini, variety True French, were grown in greenhouse facility at Department of Agricultural Science of University of Naples “Federico II” using standard horticultural practices. *C. pepo* cv. True French tissue samples were collected from young plants of about 10 cm high. Total RNA was isolated from ground, frozen leaf tissues using the SpectrumTM Plant Total RNA Kit (Sigma-Aldirch). A complete removal of traces of DNA was performed using On-Column DNase I Digest Set (Sigma-Aldirch). Quantity and integrity of the extracted total RNA were determined using NanoDrop ND-1000 Spectrophotometer (Thermo Fisher Scientific Inc., USA), on a denaturing formaldehyde gel and Agilent 2100 bioanalyzer (Agilent Technologies, USA) respectively, to be RIN > 8. Library preparation and sequencing were performed by the Genomix4Life S.r.l., spin-off of Salerno University. The sequencing library was prepared using the TruSeq RNA Sample Preparation Kit v2 (Illumina, San Diego, CA, USA) and paired-end reads of 100 bp were sequenced from the three independent samples on one lane of an illumine HiSeq 2000.

### Preprocessing and transcriptome assembly

The quality control checks on raw sequence data (75,22 millions of paired reads totalling 15 e^12^ bp) from all the three data sets was performed using FastQC (Andrews, 2010). Raw reads were filtered to remove the adapter sequences and the poorer quality regions with sequence pre-processing tool, Trimmomatic (Bolger et al., 2014). Paired-end read duplicates from the PCR amplification step in the sequencing process were removed and only those reads with a mapping score ≥ 30 were kept in the alignments. The high quality reads were aligned against the *C. pepo* reference genome sequence version 3.2 (https://cucurbigene.upv.es/) with STAR aligner (version 2.4.0j). The resulting alignment was used as input to Cufflinks (version 2.2.1) for transcript assembly. PASA pipeline (version 2.0.2) was used to combine Cufflinks results with the public transcriptome version 3.0 (https://cucurbigene.upv.es/).

### Proteome annotation and characterization of *R*-genes

The proteome functional annotation was performed through a match search against four database (TAIR10, SWISS-PROT, TrEMBL and GenBank-NR) using DIAMOND in sensitive mode with a cut-off e-value of 1 e^−5^ (Buchfink et al., 2015). To add information about protein function to our proteome, a Blast2GO (Conesa et al., 2005) annotation, using default parameters, were conducted. Finally, the zucchini proteome was scanned with InterProScan v.5.13 (Jones et al., 2014) against the InterPro protein signature databases to identify and finely characterize plant resistance proteins.

### Orthology analysis

To identify orthologous gene groups among *C. pepo, C. melo, C. sativus, C. lanatus* and *A. thaliana* we used OrthoMCL software with default settings. The association between reference *R*-genes (http://prgdb.crg.eu/) and relative orthologous group (OG) was detected using Best BLAST Hit method (BlastP, E < 1 e^−5^) and the output was filtered for a query coverage and identity percentage, both >50%.

## Results and discussion

### Transcriptome sequencing, assembly, and annotation

The sequencing produced a total of 69,5 millions of clean paired reads, obtaining 13,9 e^12^ bp of RNA-Seq data for *C. pepo* (Supplementary Table 1). Transcriptome assembly yielded 68,720 transcripts, with mean length of 1,534 bp. The transcripts were translated and a high-quality proteome of 33,966 primary protein sequences, with mean length of 316 AA, were obtained. DIAMOND similarity-based searches were performed against the publically available databases (SWISS-PROT, TrEMBL, TAIR10, and GenBank-NR) to annotate *C. pepo* proteome (Supplementary Table 2). About 85% of proteins encoded by genes had homology with four principal databases and over 75% were functionally annotated (Table 1). In addition, a GO-annotation using Blast2GO were effected and a total of 256,138 GO-terms were assigned to about 65% (21480) of the predicted proteins (Supplementary Table 3).

**Table 1:** Annotation of *C. pepo* leaf tissues transcriptome based on homology.

**Database**	**Number of DIAMOND matches**	
	**Transcriptome**	**Proteome**
SWISS-PROT	25,378 (74.7%)	21,747 (64.0%)
TrEMBL	31,640 (93.2%)	27,791 (81.8%)
TAIR10	29,885 (88.0%)	25,781 (75.9%)
GenBank-NR	31,691 (93.3%)	27,761 (81.7%)

*All transcripts and proteins were aligned against four databases and the number of transcripts and proteins with a significant hit are reported. Sensitive mode-DIAMOND matches were filtered by e-value less than 1 e-5*.

### *R*-gene annotation

**Figure 1:**
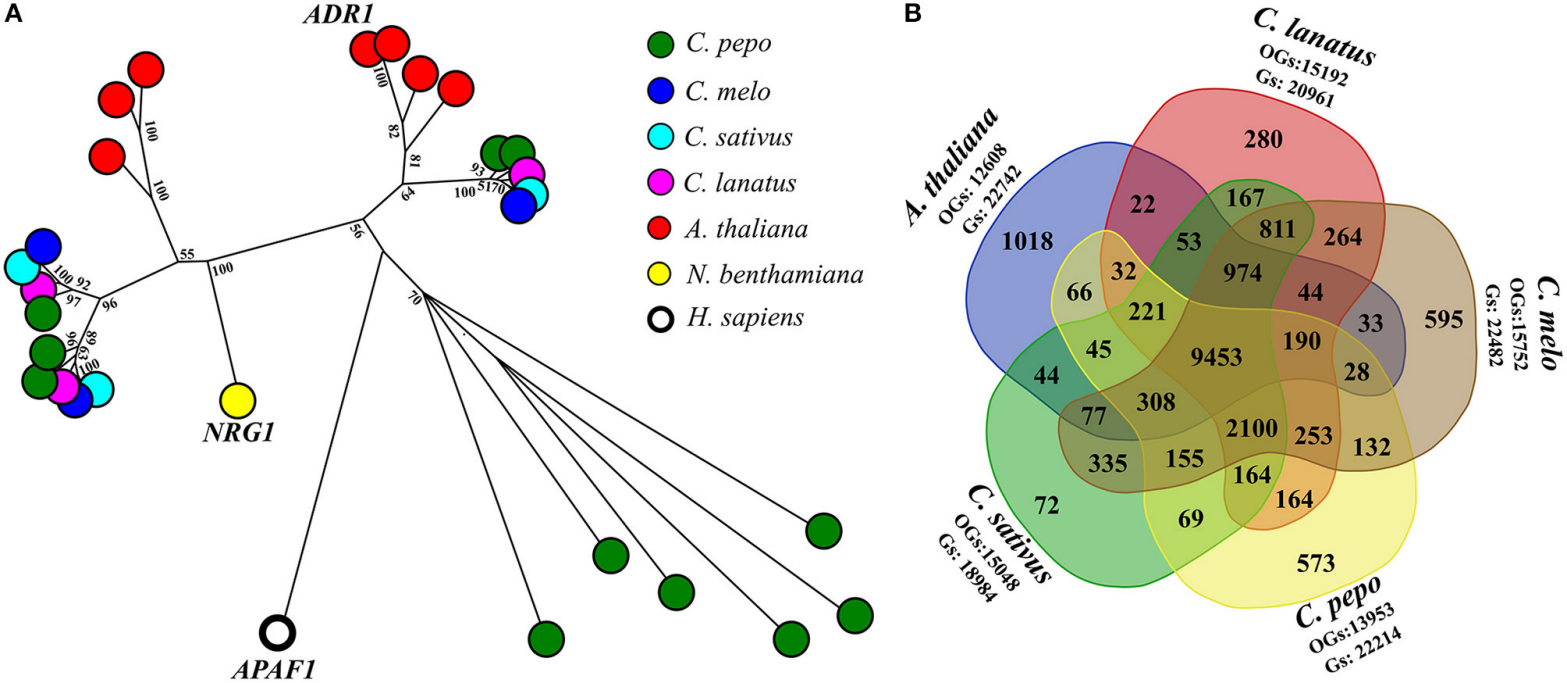
RPW8-NLR genes expansion and comparative analysis. **(A)** Evolutionary history of RPW8-NLR proteins annotated in Cucurbita pepo. Full NB domains (PF00931) of 26 RPW8-NLR proteins were used together with 3 well characterized reference genes (short gene name: ADR1, NRG1, and APAF1) to do a maximum likelihood analysis based on the Jones et al. w/freq. model. Model with the lowest BIC score (Bayesian Information Criterion) was considered to describe the substitution pattern the best. Sequence similarities were determined performing a ClustalW (default settings) multiple alignment. Evolutionary analyses were conducted in MEGA7. Labels show the bootstrap values higher than 50 (out of 100), are indicated above the branches. The tree is drawn to scale, with branch lengths proportional to the number of substitutions per site. Species to which belong sequences are indicated by colored spots. **(B)** Venn diagram of genes (Gs) clustered into orthogroups (OGs). Five species (*Cucurbita pepo, Citrullus lanatus, Cucumis melo, Cucumis sativus*, and *A. thaliana*) were used to generate the Venn diagram. In the graph were reported the number of specie-specific and common OGs.

A fine characterization of genes encoding domains similar to plant resistance (*R*) proteins, in *C. pepo* proteome was conducted. *R*-proteins can be categorized according to the presence and organization of protein domains, such as Toll/Interleukin-1 receptor (TIR), coiled coil (CC), the nucleotide-binding site (NBS), leucine-rich repeats (LRRs). A total of 64 *R*-proteins (also called NLR, NB-LRR, NBS-LRR, or NB-ARC-LRR proteins) were identified (Supplementary Table 4). The CNL (Coiled coil, Nucleotide-binding site, Leucine-rich repeats) class was divided into sub-classes based on sequence similarity with the canonical CNLs that contain an EDVID amino-acid motif, and the RPW8-like proteins (Andolfo et al., 2014b). Interestingly, an expansion of RPW8-NLR genes (11 out of 64) in *C. pepo* was discovered. Diversely, *C. melo, C. sativus*, and *C. lanatus* presented only three RPW8-NLRs for each species (Figure 1A). It is now well-known that RPW8-NLRs can function as helper NLRs for well-defined NLR–mediated resistance responses. Thus, they may enhance the *C. pepo* defense system to offset its reduced number of NRL receptors available (Sanseverino and Ercolano, 2012). In addition, *C. pepo* RPW8-NLRs showed a very high homology to *ADR1* (activated disease resistance 1), *R-*gene that confer resistance again *Erysiphe cichoracearumi*, the causal agent of Powdery Mildew (PM) in *A. thaliana* (Micali et al., 2008). PM disease, caused by *Podosphaera xanthii* (syn. *Sphaerotheca fuliginea*) has an important economic impact on *C. pepo* varieties. ADR1-like proteins expansion, identified in *C. pepo*, could suggest an adaptive diversification induced by specie-specific pathogen pressure (Andolfo and Ercolano, 2015).

### Orthologous groups

A comparative analysis among *C. pepo, C. melo, C. sativus, C. lanatus*, and *A. thaliana* were performed to obtain functional information on our proteome. A total of 18,742 orthologous groups (OGs), which included 107,386 sequences, were identified (Supplementary Table 5). 9,453 OGs enclosed 69,982 sequences and were highly conserved in all analyzed genomes (Figure 1B). A core Cucurbitaceae proteins (9,465 sequences) clustered in 2099 OGs were detected. About 65% (22,214) of *C. pepo* predicted proteins were grouped in 13,953 OGs (Figure 1B). Several *C. pepo* gene family expansions associated to transmembrane *R-*genes were discovered. One hundred zucchini proteins, annotated as Receptor-like Kinase (RLK) and Receptor-like Protein (RLP) were clustered in five OGs (OG_00004, OG_00027, OG_00038, OG_00053, OG_01889, and OG_00579) and associated a well-characterized *R*-genes (Supplementary Table 5). Probably the expansion of cell surface receptors (RLKs and RLPs) and relative strengthening of first defence line represent adaptive dynamics to balance the limited number of cytoplasmic receptors (NRLs) (Andolfo and Ercolano, 2015). The *C. pepo* gene family expansions could be associated to the cucurbit-common tetraploidization recently identified by Wang et al. (2017). Furthermore, we identified a number of gene families related to important agronomical traits. Fourteen OGs related to *OVATE* gene family grouped 19 zucchini genes. *OVATE* is an important locus for fruit shape determination and plant development (Rodríguez et al., 2011). We identified three zucchini ortholog genes to *PSY1* and *PSY3* melon genes that putatively involved in carotenoid metabolism and fruit ripening (Garcia-Mas et al., 2012). The Cup000085g037789 is the ortholog gene of *OR*, a cloned gene that governs the fruit flesh colur in melon and in other important crops (Tzuri et al., 2015). Comparative analysis of *C. pepo* proteome can be used to identify orthologous genes for functional study. Our dataset represented a very important resource to reduce the plant breeding work for the identification of candidates for important agronomical traits (Supplementary Table 5).

### Direct link to deposited data and information to users

The dataset submitted to NCBI include the raw read sequences of three biological replicates of *Cucurbita pepo* subsp. *pepo* cultivar-group Zucchini, variety True French, in FASTQ format. The raw reads of *C. pepo* can be accessed at NCBI with the following BioSample accession number: SAMN07426850 (www.ncbi.nlm.nih.gov/Traces/study/?acc=SRP114337). The *C. pepo* transcriptome annotation, in GTF format, and primary protein sequences in FASTA format can be accessed at FIGSHARE with the following link (https://figshare.com/s/8a083f60df238acdbc19). The Supplementary Material (Supplementary Tables 1–5) for this article can be found online at: (https://figshare.com/s/8a083f60df238acdbc19). Users can download and use the data freely for research purpose only with acknowledgment to us and quoting this paper as reference to the data.

## Author contributions

GA was chiefly involved in data analysis, results interpretation and manuscript writing. AD was mainly involved in data analysis, results interpretation and manuscript writing. RD drafted the manuscript. AE provided a critical reading of the manuscript. RA assembled the transcriptome. ME coordinated the project and contributed to data analysis and results interpretation. All of the authors read and approved the final manuscript.

### Conflict of interest statement

The authors declare that the research was conducted in the absence of any commercial or financial relationships that could be construed as a potential conflict of interest.

## References

[B1] Andolfo G. Ercolano M. R. (2015). Plant innate immunity multicomponent model. Front. Plant Sci. 6:987. 10.3389/fpls.2015.00987 26617626 PMC4643146

[B2] Andolfo G. Ferriello F. Tardella L. Ferrarini A. Sigillo L. Frusciante L.. (2014a). Tomato genome-wide transcriptional responses to Fusarium wilt, and tomato Mosaic virus. PLoS ONE 9:e94963. 10.1371/journal.pone.0094963 24804963 PMC4012952

[B3] Andolfo G. Jupe F. Witek K. Etherington G. J. Ercolano M. R. Jones J. D. G. (2014b). Defining the full tomato NB-LRR resistance gene repertoire using genomic and cDNA RenSeq. BMC Plant Biol. 14:120. 10.1186/1471-2229-14-120 24885638 PMC4036795

[B4] Andrews S. (2010). FastQC: A Quality Control Tool for High Throughput Sequence Data. Available online at: http://www.bioinformatics.babraham.ac.uk/projects/fastqc/

[B5] Arumuganathan K. Earle E. D. (1991). Nuclear DNA content of some important plant species nuclear DNA content material and methods. Plant Mol. Biol. Rep. 9, 208–218. 10.1007/BF02672069

[B6] Blanca J. Cañizares J. Roig C. Ziarsolo P. Nuez F. Picó B. (2011). Transcriptome characterization and high throughput SSRs and SNPs discovery in *Cucurbita pepo* (Cucurbitaceae). BMC Genomics 12:104. 10.1186/1471-2164-12-104 21310031 PMC3049757

[B7] Bolger A. M. Lohse M. Usadel B. (2014). Trimmomatic: a flexible trimmer for Illumina sequence data. Bioinformatics 30, 2114–2120. 10.1093/bioinformatics/btu170 24695404 PMC4103590

[B8] Buchfink B. Xie C. Huson D. H. (2015). Fast and sensitive protein alignment using DIAMOND. Nat. Methods 12, 59–60. 10.1038/nmeth.3176 25402007

[B9] Conesa A. Götz S. García-Gómez J. M. Terol J. Talón M. Robles M. (2005). Blast2GO: a universal tool for annotation, visualization and analysis in functional genomics research. Bioinformatics 21, 3674–3676. 10.1093/bioinformatics/bti610 16081474

[B10] Garcia-Mas J. Benjak A. Sanseverino W. Bourgeois M. Mir G. González V. M.. (2012). The genome of melon (Cucumis melo L.). Proc. Natl. Acad. Sci. U.S.A. 109, 11872–11877. 10.1073/pnas.1205415109 22753475 PMC3406823

[B11] Guo Y. L. (2013). Gene family evolution in green plants with emphasis on the origination and evolution of *Arabidopsis thaliana* genes. Plant J. 73, 941–951. 10.1111/tpj.12089 23216999

[B12] Jeffrey C. (1980). A review of the *Cucurbitaceae*. Bot. J. Linn. Soc. 81, 233–247. 10.1111/j.1095-8339.1980.tb01676.x

[B13] Jones P. Binns D. Chang H. Y. Fraser M. Li W. McAnulla C.. (2014). InterProScan 5: genome-scale protein function classification. Bioinformatics 30, 1236–1240. 10.1093/bioinformatics/btu031 24451626 PMC3998142

[B14] Micali C. Göllner K. Humphry M. Consonni C. Panstruga R. (2008). The powdery mildew disease of arabidopsis: a paradigm for the interaction between plants and biotrophic fungi. Arabidopsis Book 6:e0115. 10.1199/tab.0115 22303240 PMC3243333

[B15] Montero-Pau J. Blanca J. Bombarely A. Ziarsolo P. Esteras C. Martí-Gómez C.. (2017). *De-novo* assembly of zucchini genome reveals a whole genome duplication associated with the origin of the Cucurbita genus. *bioRxiv*. 10.1101/147702 29112324 PMC5978595

[B16] Paris H. S. (1986). A proposed subspecific classification for *Cucurbita pepo*. Phytologia 61, 133–138.

[B17] Paris H. S. (2008). Summer squash, in Vegetables I. Handbook of Plant Breeding, Vol. 1, eds Prohens J. Nuez F. (New York, NY: Springer).

[B18] Paris H. S. (2010). History of the Cultivar-Groups of *Cucurbita pepo*. Horticult. Rev. 25, 71–170. 10.1002/9780470650783.ch2

[B19] Rodríguez G. R. Munos S. Anderson C. Sim S.-C. Michel A. Causse M.. (2011). Distribution of SUN, OVATE, LC, and FAS in the tomato germplasm and the relationship to fruit shape diversity. Plant Physiol. 156, 275–285. 10.1104/pp.110.167577 21441384 PMC3091046

[B20] Sanseverino W. Ercolano M. R. (2012). *In silico* approach to predict candidate R proteins and to define their domain architecture. BMC Res. Notes 5:678. 10.1186/1756-0500-5-678 23216678 PMC3532234

[B22] Tzuri G. Zhou X. Chayut N. Yuan H. Portnoy V. Meir A.. (2015). A “golden” SNP in CmOr governs the fruit flesh color of melon (*Cucumis melo*). Plant J. 82, 267–279. 10.1111/tpj.12814 25754094

[B23] Vitiello A. Scarano D. D'Agostino N. Digilio M. C. Pennacchio F. Corrado G. (2016). Unraveling zucchini transcriptome response to aphids (No. e1635v1). PeerJ.

[B24] Wang J. Sun P. Li Y. Liu Y. Yang N. Yu J.. (2017). An overlooked paleo-tetraploidization in *Cucurbitaceae*. Mol. Biol. Evol. [Epub ahead of print]. 10.1093/molbev/msx242 29029269 PMC5850751

[B25] Wang Y. H. Behera T. K. Kole C. (eds.). (2011). Genetics, Genomics and Breeding of Cucurbits. CRC Press.

[B26] Wang Z. Gerstein M. Snyder M. (2009). RNA-Seq: a revolutionary tool for transcriptomics. Nat. Rev. Genet. 10, 57–63. 10.1038/nrg2484 19015660 PMC2949280

[B27] Wyatt L. E. Strickler S. R. Mueller L. A. Mazourek M. (2015). An acorn squash (*Cucurbita pepo* ssp*. ovifera*) fruit and seed transcriptome as a resource for the study of fruit traits in *Cucurbita*. Hortic. Res. 2:14070. 10.1038/hortres.2014.70 26504561 PMC4595981

[B28] Xanthopoulou A. Ganopoulos I. Psomopoulos F. Manioudaki M. Moysiadis T. Kapazoglou A.. (2017). *De novo* comparative transcriptome analysis of genes involved in fruit morphology of pumpkin cultivars with extreme size difference and development of EST-SSR markers. Gene 622, 50–66. 10.1016/j.gene.2017.04.035 28435133

[B29] Xanthopoulou A. Psomopoulos F. Ganopoulos I. Manioudaki M. Tsaftaris A. Nianiou-Obeidat I.. (2016). *De novo* transcriptome assembly of two contrasting pumpkin cultivars. Genom. Data 7, 200–201. 10.1016/j.gdata.2016.01.006 26981408 PMC4778644

